# Pitfall in Pupillometry: Exaggerated Ciliospinal Reflex in a Patient in Barbiturate Coma Mimicking a Nonreactive Pupil

**DOI:** 10.7759/cureus.2004

**Published:** 2017-12-30

**Authors:** Naresh Mullaguri, Nakul Katyal, Aarti Sarwal, Jonathan M Beary, Pravin George, Naresh Karthikeyan, Premkumar Nattanamai, Christopher R Newey

**Affiliations:** 1 Neurology, Cleveland Clinic Ohio; 2 Neurology, University of Missouri Columbia; 3 Neurology, Wake Forest School of Medicine; 4 Neurobehavioral Sciences, A. T. Still University

**Keywords:** ciliospinal reflex, pentobarbital, coma, status epilepticus

## Abstract

Although a neurological examination is fundamental to the evaluation of comatose patients, it is less reliable in a medically induced coma. A commonly misinterpreted finding in patients in a pentobarbital coma is altered pupillary reactivity secondary to an exaggerated ciliospinal reflex. Recognizing an exaggerated ciliospinal reflex in patients in a pentobarbital coma is important and may prevent unnecessary intervention. We present a patient induced in a pentobarbital coma for the treatment of status epilepticus who exhibited a nonreactive pupil secondary to an exaggerated ciliospinal reflex confirmed by pupillometry. We also discuss the anatomy of the ciliospinal reflex and literature regarding its clinical relevance.

## Introduction

A neurological examination is fundamental to the management of the neuro-critically ill patient. However, the reliability of this examination is diminished in circumstances such as a medically induced coma with pentobarbital. A pentobarbital coma is often used to manage refractory status epilepticus or intracranial hypertension [1]. Once a pentobarbital coma is induced, brainstem reflexes may be diminished or even lost. The ciliospinal reflex is a pupillary reflex in which the dilation of the pupil follows ipsilateral cervicocranial noxious stimulation [2-3]. It is typically enhanced in coma or sleep. Although other brainstem reflexes may diminish or even cease during a pentobarbital coma, the ciliospinal reflex can stay pronounced despite the barbiturate. Patients may demonstrate anisocoria with poor reactivity that can mimic a nonreactive pupil, indicating a neurologic emergency [2-3]. We report a case of a persistent nonreactive pupil during noxious stimulation in a patient with refractory status epilepticus requiring a pentobarbital coma. We present a review of the literature on the exaggerated response of the ciliospinal reflex in a barbiturate coma.

## Case presentation

**Figure 1:**
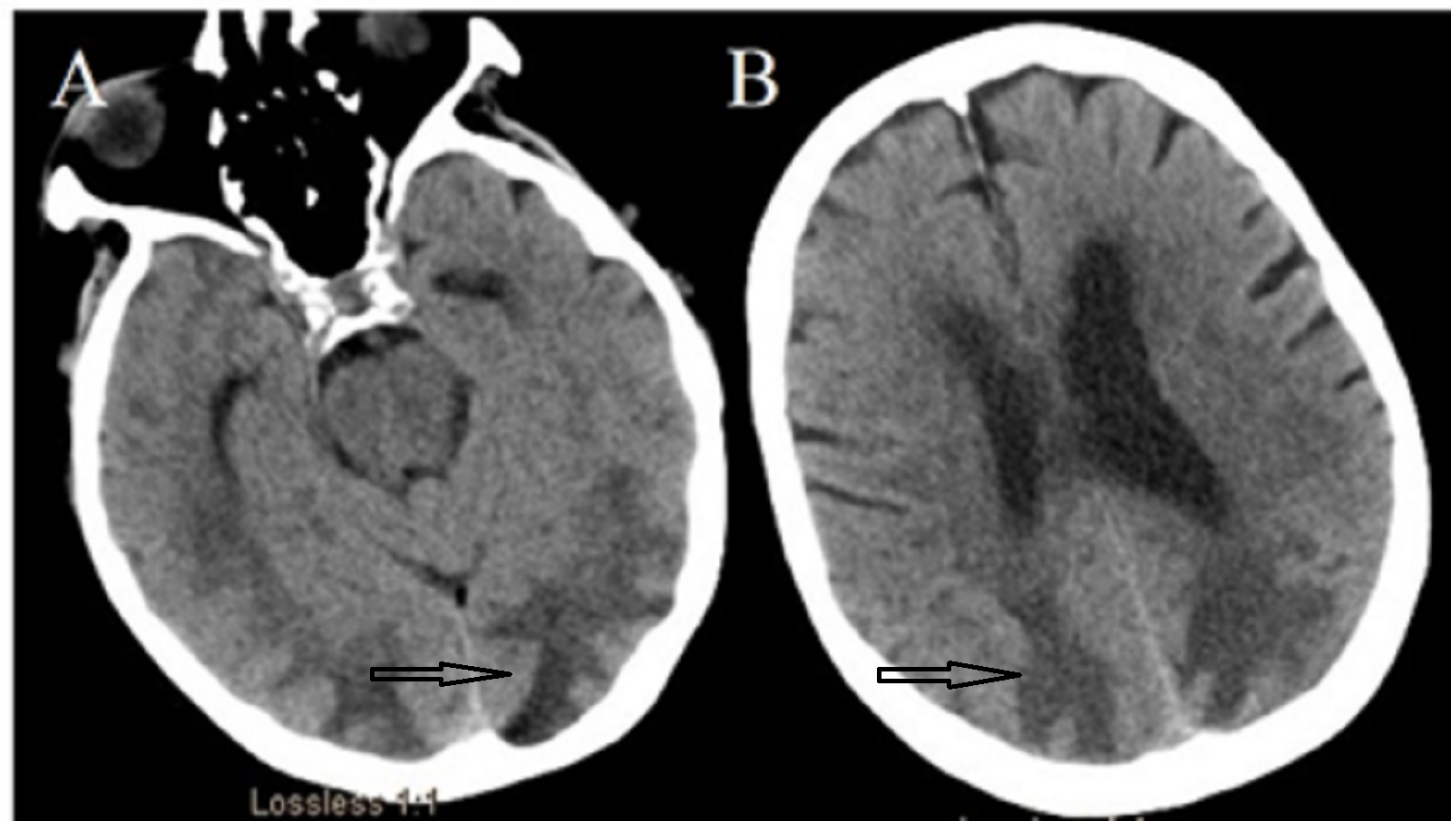
Computed tomography (CT) of the head without contrast CT showing bilateral posterior quadrant hypodensities consistent with vasogenic edema. These are typical radiological findings in posterior reversible encephalopathy syndrome (PRES).

A 76-year-old Caucasian female presented with a history of methicillin-resistant Staphylococcus aureus necrotizing pneumonia and empyema of the right lower lung. She underwent a lobectomy with a prolonged hospital course in the medical intensive care unit eventually complicated by a prolonged, generalized tonic-clonic seizure requiring intubation. Her seizures were refractory to three antiepileptic medications (levetiracetam, lacosamide, and fosphenytoin) and continuous midazolam infusion (maximum rate of 40 mg/h). A computed tomography (CT) scan of the head showed extensive vasogenic edema in the bilateral hemispheres, indicating posterior reversible encephalopathy syndrome (PRES), as shown in Figure 1A and Figure 1B.

**Figure 2:**
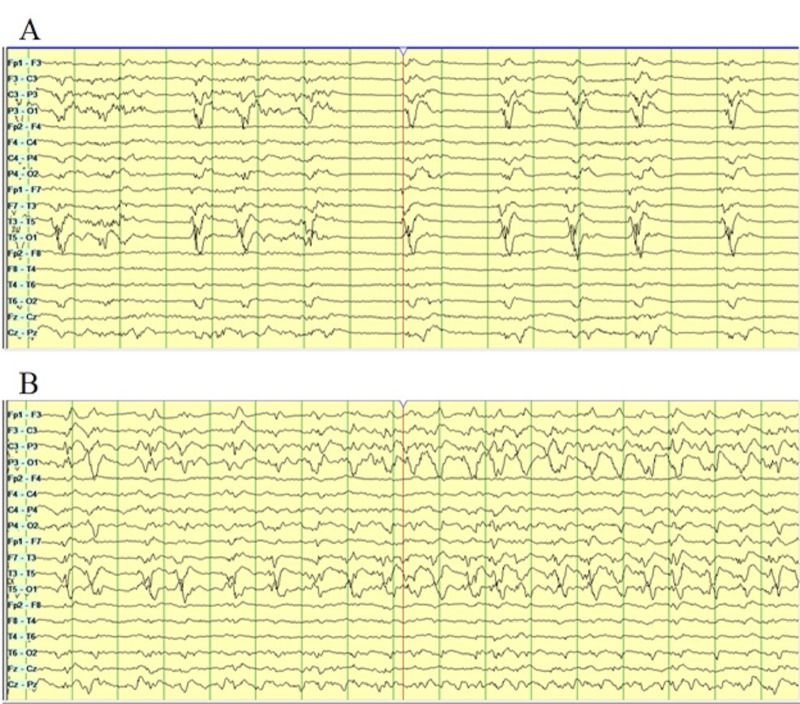
Continuous electroencephalography (CEEG) Bi-temporal montage on CEEG showing diffuse slowing with lateralized periodic discharges (LPDs) with over-riding beta activity in the left occipital region (A). Electrographic seizures evolving from the left posterior quadrant on CEEG (B).

Continuous electroencephalographic monitoring (CEEG) showed left occipital lateralized periodic discharges (LPDs; Figure 2A), evolving into subclinical electrographic seizures (Figure 2B).

Her seizures remained refractory, requiring an escalation of the treatment to a medically induced coma with pentobarbital. After 24 hours of burst suppression, the patient’s left pupil became nonreactive to brief penlight stimulation as well as pupillometry (Neuroptics, Irvine, CA, USA; Table 1).

**Table 1:** Pupillometer values The pupillometer shows a sluggishly reactive right pupil and a nonreactive left pupil. Abbreviations: NPI, neurological pupil index; MIN, minimum; CH, change; CV, constriction velocity; MCV, mean constriction velocity; LAT, latency; DV, dilation velocity; mm, millimeters; s, second.

	Right Pupil	Left Pupil
NPI	2.2	0
Size	3.4 mm	3.22
MIN	3.33	_
CH	2%	_
CV	0.05 mm/sec	_
MCV	0.31 mm/sec	_
LAT	0.33 sec	_
DV	0.02 mm/sec	_

**Figure 3:**
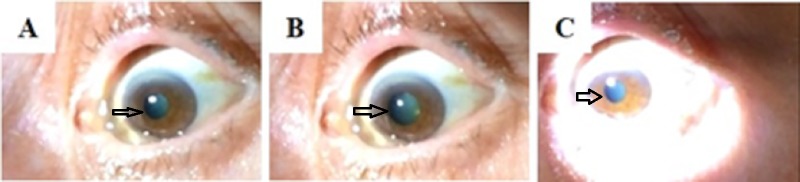
Pupillary light reflex A “nonreactive” left pupil to penlight stimulation (A). After 15 seconds of noxious stimulation to the ipsilateral cervical region, there is dilation of the pupil (B). This demonstrates an intact ciliospinal reflex. Penlight stimulation was then applied for 23 seconds, resulting in pupillary constriction (C).

Her right pupil was sluggishly reactive. Mydriasis was noted to be elicited with noxious stimulation to the ipsilateral neck and resolved after 23 seconds of prolonged penlight stimulation (Figure 3A to Figure 3C).

**Figure 4:**
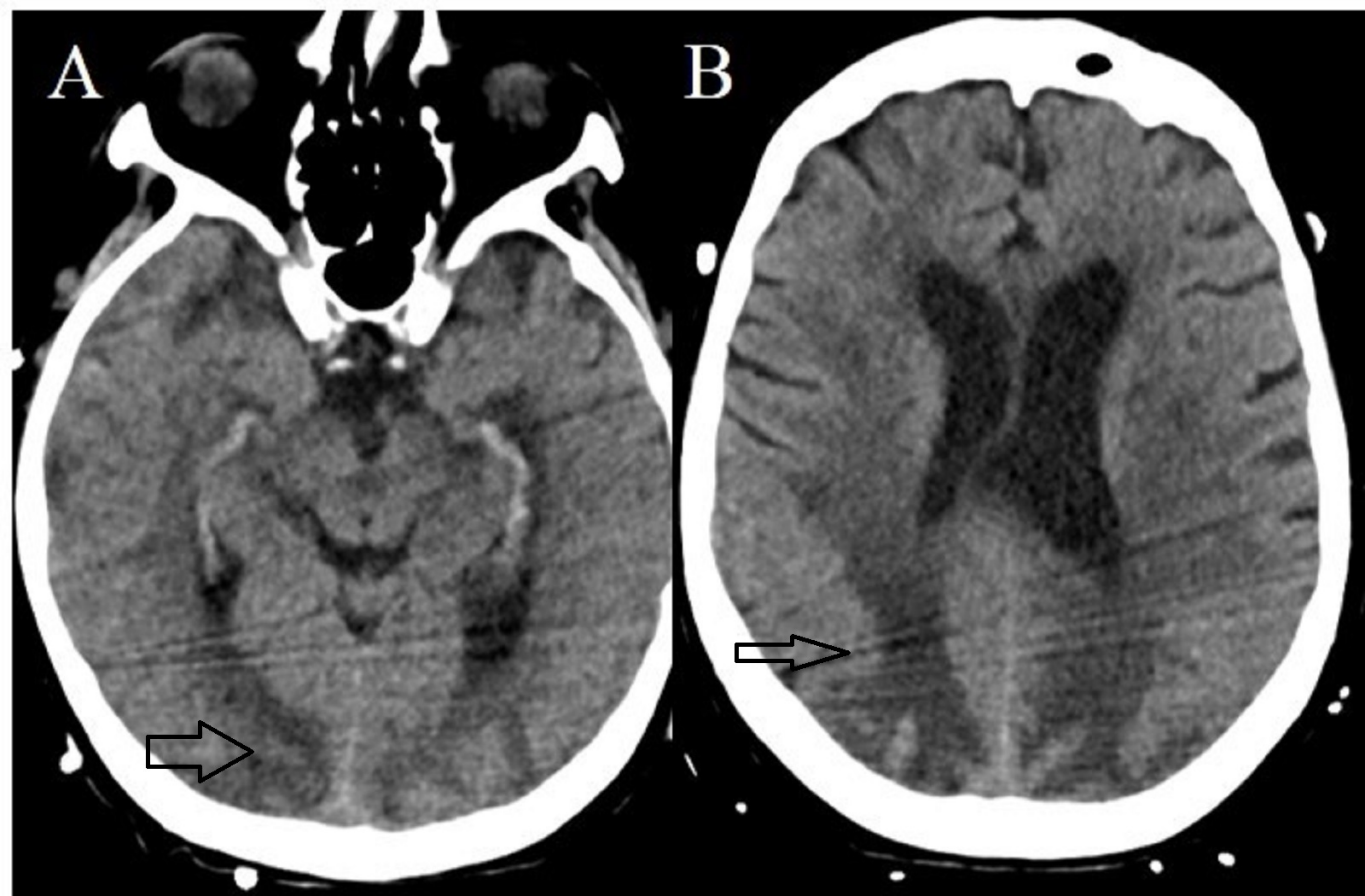
Computed tomography (CT) of the head without contrast There is hyperdensity within the bilateral posterior quadrants consistent with hemorrhagic transformation.

A repeat CT head was initially deferred. A CT head three days after the initial CT (24 hours after the noted pupillary changes) showed evolving changes of the known PRES (Figure 4A and Figure 4B) but no progression of cerebral herniation.

She was eventually weaned off pentobarbital over the next few days. Her bedside pupillary function normalized. Unfortunately, the patient developed multisystem organ failure, and after a goals of care discussion, the family transitioned the patient to comfort care.

## Discussion

Our case highlights the phenomenon of an exaggerated ciliospinal reflex in a patient in a pentobarbital coma mimicking a nonreactive pupil that could raise concern for a neurological emergency. Recognizing this reflex affected the decision for neuroimaging and prevented the patient from undergoing unnecessary testing. We also provide a confirmation of the reflex via pupillometry.

The ciliospinal reflex is a self-limited reflex pupillary dilation induced by noxious stimuli to the face, neck, or upper trunk [2-3]. The afferent limb of the reflex is mediated by trigeminal or cervical pain fibers, and the efferent limb is mediated by sympathetic fibers from the lower cervical and upper thoracic spinal cord [4]. The central sympathetic fibers (i.e., first-order neurons) of this reflex originate in the hypothalamus and descend through the brainstem in close proximity to the trigeminal nucleus into the cervical spinal cord through the upper thoracic segments [4]. The sympathetic fibers leave the cervicothoracic cord (C8-T2; i.e., second-order neurons) through the dorsal spinal root to enter the paravertebral sympathetic chain and ultimately the superior cervical ganglion [4]. The third-order neurons ascend through the arterial system on the internal and external carotid arteries. The pupil receives sympathetic innervation from the sympathetic fibers of the ophthalmic artery, which is a branch of the internal carotid artery [4]. The ciliospinal reflex can elicit the sympathetic innervation of the pupillary reflex, bypassing the first-order neurons. Local cutaneous stimulation of the neck activates sympathetic fibers via connections with the ciliospinal center at C8-T2 [2-4]. The induction of a pentobarbital coma is expected to cause a suppression of all brainstem reflexes, which can obscure the clinical signs of neurological deterioration [5-6]. In some cases, the ciliospinal reflex may not be suppressed in a barbiturate coma and can become exaggerated. Thus, the pupillary light reflex and pupillometry is less reliable and can mimic a neurological emergency [6]. The ciliospinal reflex in patients who are in a pentobarbital coma has been previously described by Andrefsky et al. These authors reported an exaggerated ciliospinal reflex following head and neck repositioning [6]. These maneuvers caused pupillary dilation that persisted for 1- 6 minutes [6]. Our case adds to this literature and highlights the importance of recognizing the ciliospinal reflex, particularly in neuro-critically ill patients in a pentobarbital coma.

Timely and accurate recognition of an exaggerated ciliospinal reflex in a vulnerable population is very important. It should be identified promptly and confirmed with prolonged pupillary light stimulation, not with pupillometry, as we have demonstrated. The prolonged light stimulation test can readily differentiate this phenomenon from serious underlying neurological conditions, such as from midbrain lesions and oculomotor nerve palsy secondary to increased intracranial pressure or posterior communicating artery aneurysms [5-6]. Pupillary dilation in such conditions remains unaffected by prolonged light stimulation [6].

## Conclusions

The recognition of the ciliospinal reflex in a pentobarbital-induced coma is important and an awareness of an exaggerated response mimicking a nonreactive pupil is also important and may avoid unnecessary diagnostic testing.
